# Sexual health of female breast cancer survivors and their partners

**DOI:** 10.1007/s00404-026-08314-5

**Published:** 2026-01-22

**Authors:** Anna Sophia Flechtenmacher, Lina Judit Schiestl, Susanne Singer, Annette Hasenburg

**Affiliations:** 1 Department of Dermatology, Venereology and Allergology, Medical Faculty Mannheim, Ruprecht-Karls-University Heidelberg, Mannheim, Germany; 2 Department of Obstetrics and Gynecology, University Medical Center, Johannes Gutenberg University Mainz, Mainz, Germany; 3 Department of Quality of Life in Oncology, Comprehensive Cancer Center Mecklenburg-Vorpommern (CCC-MV), University Medical Center, Rostock, Germany

**Keywords:** EORTC QLQ-C30, EORTC QLQ-SH22, Patient-reported outcomes, Sexual satisfaction, Sexuality

## Abstract

**Purpose:**

To investigate sexual health in breast cancer survivors and their partners, focusing on sexual satisfaction, changes in satisfaction with partner sexuality before vs. after the diagnosis, and unmet informational needs on sexual health in the context of breast cancer.

**Methods:**

Breast cancer patients and their partners were surveyed at a single time point 1–5 years after having completed primary therapy for breast cancer as part of a cross-sectional study. Study participants completed self-report-questionnaires covering personal characteristics, a question on satisfaction with partner sexuality before (retrospective assessment) versus after the diagnosis (based on the Sexual Medicine Questionnaire for Chronic Diseases (SFCE)), the EORTC (European Organisation for Research and Treatment of Cancer) Sexual Health Questionnaire (EORTC QLQ-SH22) together with the EORTC Quality of Life Core Questionnaire (EORTC QLQ-C30), and a questionnaire assessing sexual health care. Descriptive statistics were used to summarize demographic and clinical data. For group comparisons, dyadic dependencies were accounted for, applying paired *t* tests when normality (Shapiro–Wilk test) was met and Wilcoxon signed-rank test otherwise.

**Results:**

A total of 128 participants (64 patients, 64 partners) were enrolled. Sexual satisfaction did not differ between patients (*M* = 55, SD = 20.9) and partners (*M* = 56.7, SD = 20) (*t* test, *p* = 0.46). A positive correlation was found between patients’ and partners’ sexual satisfaction (*r* = 0.62, *p* < 0.0001). Satisfaction with partner sexuality was lower after diagnosis (*p* < 0.001, *r* = 0.54)—with both patients and partners being less satisfied after the diagnosis (M = 2.58, SD = 0.95) than before (*M* = 3.14, SD = 0.74). Overall, 75% of the study participants reported not having received information about sexual health issues related to breast cancer, while 64% expressed a desire for more information.

**Conclusion:**

The findings of this study highlight the importance of considering couple dynamics in breast cancer care. Patients and partners have unmet needs concerning sexual health in the context of breast cancer. Addressing sexuality may improve quality of life and psychosocial adjustment. Future research should include larger, more diverse samples and focus on assessing sexuality and sexual health as multidimensional constructs in line with WHO (World Health Organization) definitions.

## What does this study add to the clinical work

**Table:** 

This study indicates that breast cancer diagnosis and treatment impact satisfaction with partner sexuality and highlights the interdependence between patients’ and partners’ sexual satisfaction. It underlines the need to address sexuality and relationship dynamics in breast cancer care, as many patients and partners report unmet informational needs.	

## Introduction

With approximately 2,3 million new cases annually worldwide, breast cancer is the most common malignancy among women [1]. As survival rates increase due to advances in diagnostics and treatment, quality of life has become an increasingly important focus in both clinical care and research [2].

Sexuality and sexual health are integral aspects of quality of life. They have been defined by the World Health Organization (WHO) as multidimensional constructs influenced by biological, psychological, social, and cultural factors [3]. Breast cancer diagnosis and treatment can profoundly affect patient’s sexuality and sexual health. Prevalence estimates for sexual dysfunction among breast cancer patients range from 30 to 100% [4, 5]. For most women the female breast is symbolically and functionally highly significant [6]. Alterations due to cancer treatment can challenge a woman’s sense of femininity and lead to reduced self-esteem, feelings of unattractiveness, shame, and sexual withdrawal [7]. Surgical treatment may lead to asymmetry, fibrosis, scarring, lymphedema, sensory loss, and pain in the breast area [8–10]. Radiotherapy may moderately affect sexual function due to fatigue, sleep disturbances, and pain [11, 12]. Chemotherapy is a key risk factor for sexual dysfunction, particularly regarding arousal, lubrication, orgasm, and pain during intercourse [2, 13]. The toxicity of chemotherapy can lead to mucosal damage, alopecia, nausea, fatigue, and ovarian insufficiency potentially resulting in chemotherapy-induced menopause, amongst others [14, 15]. Endocrine therapies are associated with various adverse effects which can affect patients’ sexual well-being, e.g., hot flashes, reduced sexual desire, weight gain, musculoskeletal pain, depression, cognitive impairment, and fatigue [16, 17]. In premenopausal women, these effects may be even more pronounced due to abrupt estrogen suppression [18].

Despite its significance, sexuality, and sexual health in the context of breast cancer often remain unaddressed in clinical practice partly due to being a taboo topic [19, 20]. Communication barriers may also include healthcare providers’ lack of time and felt incompetence in talking about it and offering support [21]. Partners are often excluded from discussions about the impact of breast cancer on sexuality [22, 23]. However, research indicates that the impact of breast cancer diagnosis and treatment extends beyond the patient, affecting intimate relationships and partners, who often struggle with emotional and communicative challenges [24–27]. Changes in intimacy, sexual activity, and relationship dynamics in the context of breast cancer have been documented in previous studies [27–29]. Transitioning from the roles of patient and caregiver back to sexual partners can be difficult, and communication about sexuality is frequently avoided due to fear, insecurity, or lack of communication skills [29, 30]. Persistent communication barriers may negatively affect relationship quality [27]. However, open communication, mutual support, and shared coping can strengthen relationships, foster emotional closeness, and improve psychological well-being [25, 31, 32]. While some couples experience strain, others report strengthened bonds through mutual support [25, 28, 29].

Despite the recognized importance of sexual health, many breast cancer patients and their partners report insufficient guidance from healthcare professionals [33, 34].

This study aimed to assess problems with sexuality and sexual health among breast cancer survivors and their partners in Germany. The focus was on the following three key aspects: sexual satisfaction, perceived changes in satisfaction with partner sexuality before vs. after breast cancer diagnosis, and unmet needs for information on sexuality and sexual health in the context of breast cancer.

## Methods

### Study design and procedures

**Fig. 1:**
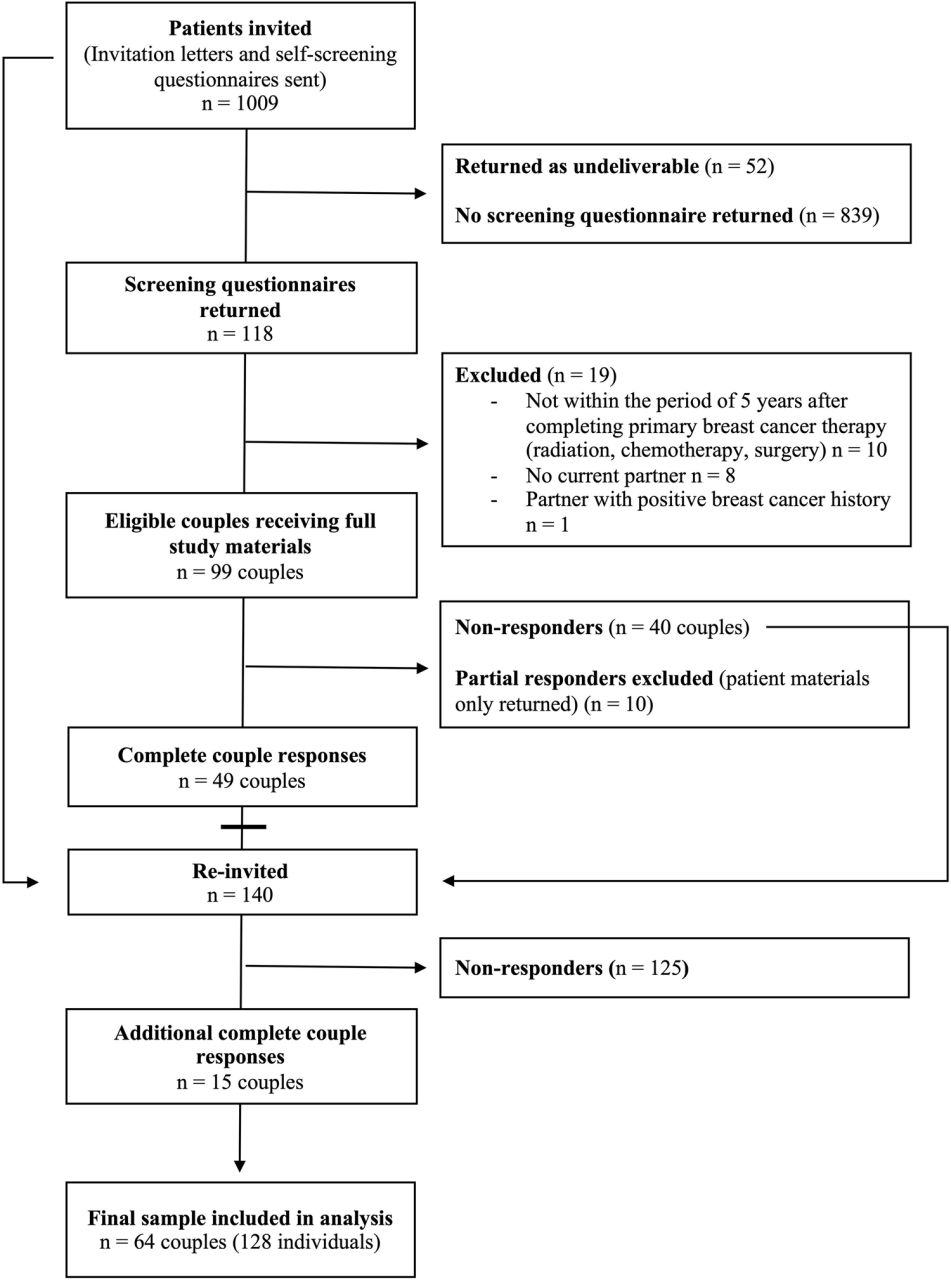
Participant flow through the study

A cross-sectional study was initiated by the Clinic for Obstetrics and Women’s health of the University Medical Center Mainz, Germany. Two groups were recruited: Breast cancer survivors (Group 1) and their partners (Group 2). Group 1 included women aged between 18 and 80 years who had undergone surgery for primary breast cancer between January 2017 and June 2022 at the University Medical Center Mainz. They were required to be within five years of completing primary breast cancer treatment (surgery, radiotherapy, chemotherapy) and in a romantic relationship. Group 2 consisted of their romantic partners (any gender), aged at least 18 years, without a personal history of breast cancer. All eligible (former) breast cancer patients (*N* = 1008) received a postal invitation with study information and a screening questionnaire to assess eligibility. Women interested in participating returned the completed screening form. Only after this form was returned were full study materials sent to the couples that fulfilled the inclusion criteria stated above (*N* = 95 couples). The participants were asked to return the completed questionnaires and signed consent forms in stamped return envelopes. Participants were explicitly instructed in the invitation letter and study invitation to complete the questionnaires independently. To ensure this, each couple received a separate set of study materials – one version of the patient and one for the partner—together with two different return envelopes. Data collection was conducted from February 2023 to September 2023. Reminder letters were sent once in July 2023, due to limited recourses only 140 in number. See Fig. 1 for detailed participant flow through the study.

### Instruments

Questionnaire sets were created for the two Groups. In the respective section, sociodemographic, medical and lifestyle characteristics were collected. Sexual satisfaction was assessed using the “Sexual Satisfaction” scale of the European Organisation for Research and Treatment of Cancer (EORTC) Sexual Health Questionnaire (EORTC QLQ-SH22) [35], with higher scores indicating greater satisfaction. The QLQ-SH22 is a multidimensional measure of sexual health in cancer patients applicable across genders and sexual orientations. The questionnaire was psychometrically validated showing acceptable to high internal consistency (Cronbach’s *α* = 0.80–0.90), and a test–retest reliability ranging from *r* = 0.70 to *r* = 0.93 [35]. Quality of life was assessed using the global health/quality of life scale of the EORTC Quality of Life Core Questionnaire (EORTC QLQ-C30) [36]—a widely used instrument for assessing quality of life in cancer patients. Its psychometric framework and quality criteria have been evaluated in the international field study my Aaronson et al. [36], demonstrating good internal consistency for most multi-item scales (Cronbachs’s *α* ≥ 0.70), a supported scale structure, and clinical validity. Satisfaction with partner sexuality (before and after diagnosis) was assessed by a single question based on the "Sexual Medicine Questionnaire for Chronic Diseases" (SFCE) by Ahlers et al. [37, 38]. The original question (“How satisfied were you/are you with your sexuality”) was modified by the adding the term “partner sexuality” to specifically address sexual interaction within the couple relationship. The inclusion of this item was methodologically justified because, unlike the EORTC QLQ-SH22, the SFCE is designed to collect sexual health information at two distinct time points (“before diagnosis” and “after diagnosis”). This structure allows a quasi-longitudinal comparison within a cross-sectional study design—an essential component for our study aim to examine perceived changes in satisfaction with partner sexuality. As the SFCE has not been formally validated, this item was used solely as an exploratory indicator of perceived change rather than as a psychometrically robust outcome measure. Questions about the care situation were taken from SFCE [37, 38].

### Statistical analysis

Statistical analyses were conducted using R statistical software (version 4.3.2). Frequencies, percentages, means, standard deviations (SD), and quartiles were used to describe the characteristics of the participants. Given the dyadic nature of the relationships studied, patients and partners were treated as dependent samples in group comparisons. Accordingly, inferential statistical methods for dependent samples were applied. Prior to each comparison, the assumption of normally distributed differences required for paired *t* tests was assessed using the Shapiro–Wilk test. If the assumption was met, paired *t* tests were used; if not, the non-parametric Wilcoxon signed-rank test was applied. To examine the association between the sexual satisfaction of breast cancer patients and that of their partners, the Pearson correlation coefficient was computed. Missing data were handled according to the EORTC scoring manual, allowing computation of scale scores when at least 50% of items were completed. For the single item from the SFCE, analyses were conducted using available data only (pairwise deletion). No imputation procedures were allied.

### Ethical consideration

The study was approved by the Ethics Committee of the State Medical Association of Rhineland-Palatinate on October 13, 2022 (Protocol No. 2022-16713_1). The legal basis for data processing in this study was the informed consent of participants in accordance with Article 6(1) of the General Data Protection Regulation (GDPR).

### Use of large language models

The manuscript was proofread by ChatGPT-5 (OpenAI; USA) to increase readability. Following the utilization of these tools, the authors examined and revised the content as required, assuming complete responsibility for the publication’s material.

## Results

Data were collected from 128 individuals (64 breast cancer patients, 64 partners). The response rate to the invitation letters was 9%; 67% of those who received the full study materials completed and returned them.

### Participant characteristics

Breast cancer patients were 58.4 years old on average (SD = 11.1, min 36, max 77), their partners 61.8 years (SD = 11.4, min 30, max 81). The sample consisted almost exclusively of heterosexual, cisgender couples with high education and financial status (Table 1). 82% of breast cancer patients had undergone breast-conserving surgery; 17% mastectomy. None of them reported recurrence or distant metastases after the diagnosis (Table 2).

**Table 1:** Demographic characteristics of the sample

	Total sample	Breast cancer patients	Partners
Individuals *N* (%)	128 (100)	64 (50)	64 (50)
Gender identification *N* (%)			
Female	63 (49.2)	63 (98.4)	63 (98.4)
Male	63 (49.2)	0 (0)	0 (0)
No answer	2 (1.6)	1 (1.6)	1 (1.6)
Age in years			
Mean (SD)	60.1 (11.3)	58.4 (11.1)	61.8 (11.4)
Median (1st Quartile, 3rd Quartile)	61 (51, 69)	59 (50.5, 67.5)	64 (54, 70.5)
Highest level of education *N* (%)			
Primary/secondary school	19 (14.8)	7 (10.9)	12 (18.8)
Intermediate/secondary School/10th grade	23 (18.0)	17 (26.6)	6 (9.4)
University of applied sciences	15 (11.7)	6 (9.4)	9 (14.1)
High/ upper secondary school	68 (53.1)	33 (51.6)	35 (54.7)
Other/no answer	3 (2.3)	1 (1.6)	2 (3.1)
Financial Situation *N* (%) (How well do you manage with the money available to you each money?)			
Very poor	1 (0.8)	1 (1.6)	0 (0)
Poor	7 (5.47)	4 (6.3)	3 (4.7)
Good	66 (51.6)	30 (46.9)	36 (56.3)
Very good	52 (40.6)	28 (43.7)	23 (35.9)
No answer	3 (2.3)	1 (1.6)	2 (3.1)
How strongly do religious beliefs influence your daily life, especially your sexuality and partnership? *N* (%)			
Not at all	94 (73.4)	45 (70.3)	49 (76.6)
Slightly	22 (17.2)	11 (17.2)	11 (17.2)
Strongly	8 (6.3)	5 (7.8)	3 (4.7)
Very strongly	1 (0.8)	1 (1.6)	0 (0)
No answer	2 (1.6)	1 (1.6)	1 (1.6)
Family planning completed *N* (%)			
Yes	122 (95.3)	61 (95.3)	61 (95.3)
No	5 (3.9)	2 (3.1)	3 (4.7)
No answer	1 (0.8)	1.6)	0 (0)
Postmenopausal			
Yes	–	55 (85.9)	–
No	–	6 (9.4)	–

**Table 2:** Breast cancer specific medical history

TNM stage	Breast cancer patients *N* = 64
	*N* (%)
Dignity	
Invasive	54 (84.4)
Ductal Carcinoma in Situ (DCIS)	10 (15.6)
T stage	
Tis	10 (15.6)
T1	36 (56.3)
T2	17 (26.6)
T3	1 (1.6)
N stage	
NX	9 (14.1)
N0	41 (64.1)
N1	11 (17.2)
N2	2 (3.1)
N3	1 (1.6)
M stage	
M0	64 (100)

### Quality of life

Breast cancer patients reported a mean Global Health/Quality of life score of 67.1 (SD = 18.9), while partners scored 73.9 (SD = 14.3) (score range 0–100) (Table 3), indicating slightly higher well-being among partners.

**Table 3:** Quality of life

	Breast cancer patients *N* = 64	Partners *N* = 64
	Mean (SD)	Mean (SD)
EORTC QLQ-SH22 Importance of Sexual Activity *	59.1 (27.3)	64.0 (28.5)
EORTC QLQ-SH22 Communication with Professionals ^*^	14.8 (24.5)	5 (14.8)
EORTC QLQ-SH22 Confidence Erection *	–	66.7 (33.3)
EORTC QLQ-SH22 Sexual Satisfaction *	55.0 (20.9)	56.7 (12.0)
EORTC QLQ-SH22 Decreased Libido ^**#**^	57.9 (34.9)	28.5 (26.2)
EORTC QLQ-SH22 Incontinence ^**#**^	17.5 (29.2)	9.1 (19.2)
EORTC QLQ-SH22 Fatigue ^**#**^	48.7 (34.3)	24.7 (26.9)
EORTC QLQ-SH22 Treatment ^**#**^	49.2 (35.7)	25.8 (37.4)
EORTC QLQ-SH22 Partnership (insecurity about ability to satisfy partner) ^**#**^	32.2 (33.9)	29.4 (32.8)
EORTC QLQ-SH22 Body Image (female) ^**#**^	17.5 (28.0)	–
EORTC QLQ-SH22 Vaginal Dryness ^**#**^	55.2 (36.4)	–
EORTC QLQ-SH22 Sexual Pain ^**#**^	29.4 (29.6)	10.0 (15.3)
EORTC QLQ-C30 Global Health/Quality of Life *	67.1 (18.9)	73.9 (14.3)

^*^ denotes function scales, with high scores representing good quality of life (Range: 0–100)

^*#*^ denotes symptom scales, with high scores representing poor quality of life (Range: 0–100)

### Sexual satisfaction

**Fig. 2:**
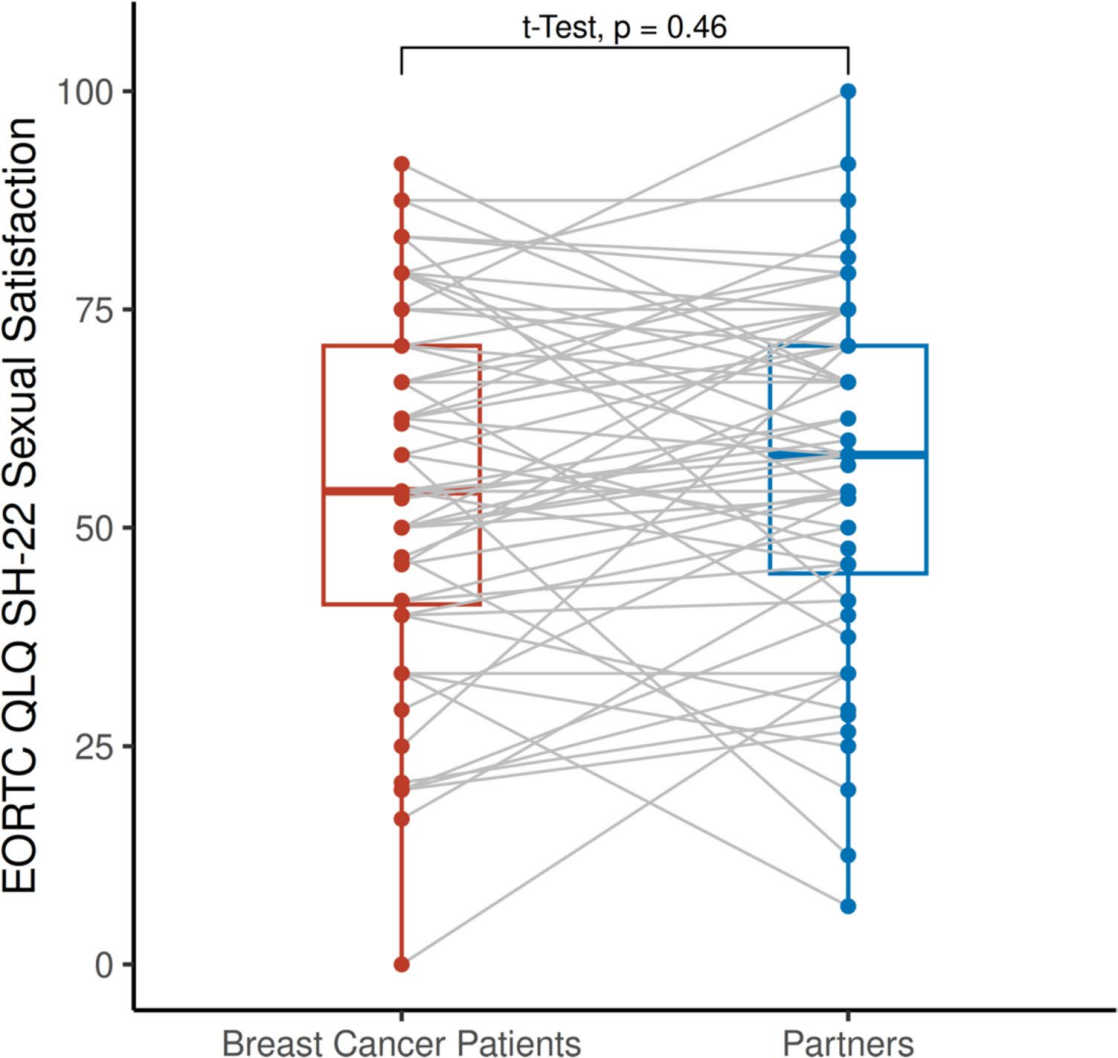
Sexual Satisfaction in Breast Cancer Patients and their Partners. *EORTC* European Organisation for Research and Treatment of Cancer. *QLQ* Quality of Life Questionnaire. *SH-22* Sexual Health Questionnaire

**Fig. 3:**
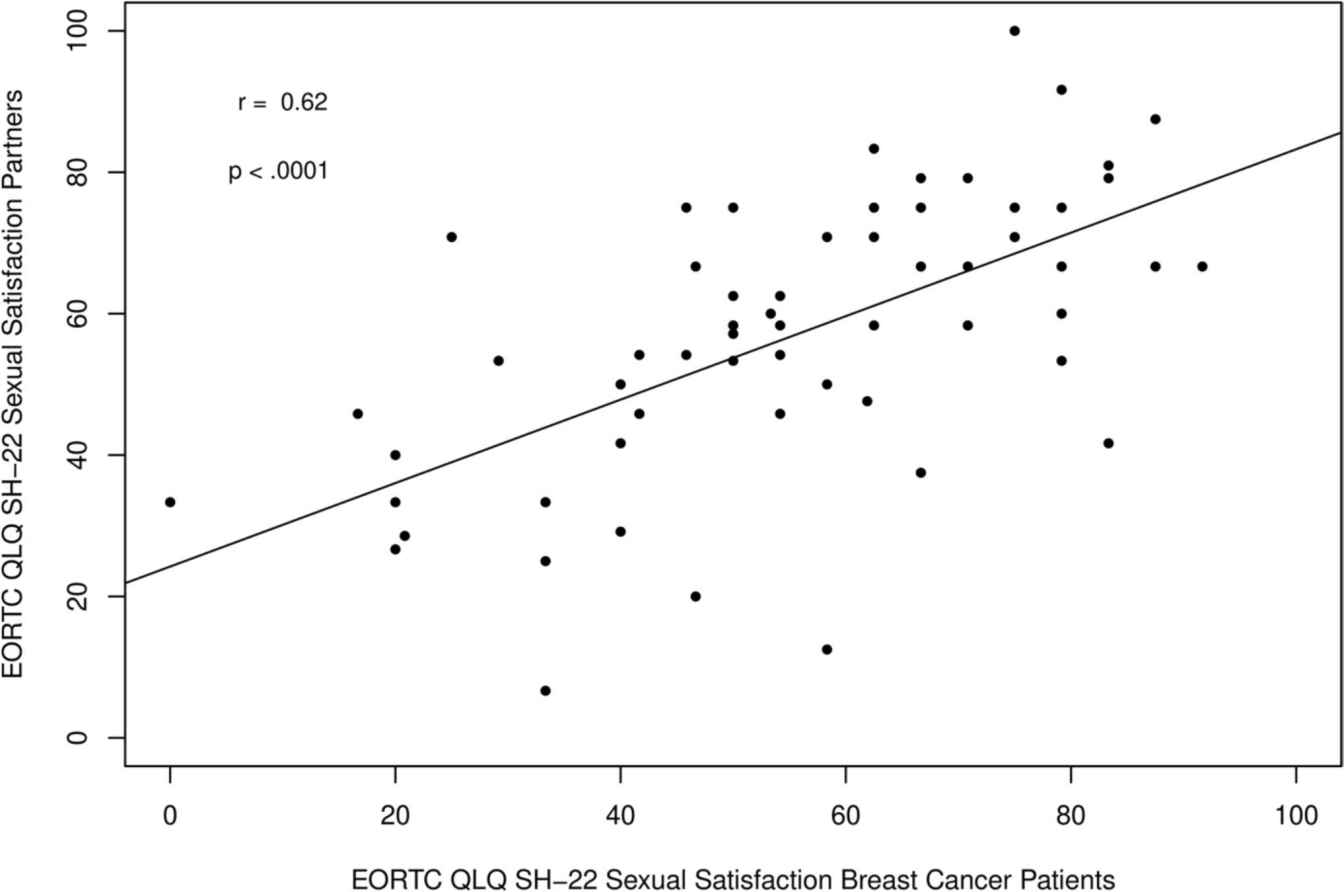
Association of Sexual Satisfaction in breast cancer patients and their partners. *EORTC* European Organisation for Research and Treatment of Cancer. *QLQ* Quality of Life Questionnaire. *SH-22* Sexual Health Questionnaire

Mean EORTC QLQ-SH22 sexual satisfaction scores (score range 0–100) were comparable between breast cancer patients (M = 55.0, SD = 20.9) and partners (M = 56.7, SD = 20.0) (Table 3), with no statistically significant difference (mean difference = 1.7, 95% CI − 6.3 to 2.9, *p* = 0.46) (Fig. 2). A moderate-to-strong positive correlation was observed between patients’ and partners’ sexual satisfaction (Pearson’s *r* = 0.62, *p* < 0.0001, *R*^2^ = 0.38, power = 99.9%), indicating dyadic interdependence (Fig. 3).

### Satisfaction with partner sexuality before vs. after breast cancer diagnosis

Across the entire sample, satisfaction with partner sexuality declined after diagnosis (M_before = 3.14, SD = 0.74; M_after = 2.58, SD = 0.95) when measured with a single, partly retrospective item based on the SFCE. This difference was statistically significant and corresponded to a medium effect size (mean difference = 0.56, 95% CI 0.40–0.72, *p* < 0.001, *r* = 0.54). There was no difference in this decline between breast cancer patients and partners (*p* = 0.63) (See Table 4).

**Table 4:** Information received on sexuality-related concerns in the context of breast cancer

	Total sample *N* = 128	Breast cancer patients *N* = 64	Partners *N* = 64
Have you received information about possible sexual problems related to breast cancer? *N* (%)			
Yes	26 (20.3)	14 (21.9)	12 (18.8)
No	96 (75)	47 (73.4)	49 (76.6)
Missing	6 (4.7)	3 (4.7)	3 (4.7)
How satisfied where you with the amount of the information received? *N* (%)			
Not at all	45 (35.2)	23 (35.9)	22 (34.4)
A little	36 (28.1)	20 (31.3)	16 (25)
Quite a bit	29 (22.7)	13 (20.3)	16 (25)
Very much	4 (3.1)	1 (1.6)	3 (4.7)
Missing	14 (10.9)	7 (10.9)	7 (10.9)
Would you like to receive more information about sexuality in the context of breast cancer in the future? *N* (%)			
Yes	82 (64.1)	44 (68.8)	38 (59.4)
No	40 (31.3)	17 (26.6)	23 (35.9)
Missing	6 (4.7)	3 (4.7)	3 (4.7)
If yes, who would you prefer to receive this information from? *N* (%)			
Physicians	77 (60.2)	41 (64.1)	36 (56.3)
Psychologists	43 (33.6)	27 (24.1)	16 (25)
Pharmacists	2 (1.6)	2 (3.1)	0 (0)
Nurses	4 (3.1)	3 (4.7)	1 (1.6)
Other professional caregivers	3 (2.3)	3 (4.7)	0 (0)
Others	3 (2.3)	2 (3.1)	1 (1.6)
Missing	41 (32.0)	18 (28.1)	23 (35.9)

### Healthcare provision concerning sexuality following breast cancer

A substantial proportion of participants (75%) reported not having received information on potential sexual problems related to breast cancer. Despite this, 64% expressed a desire for more information on sexuality in the context of breast cancer, preferably from medical or mental health care professionals, indicating a pronounced unmet informational need.

## Discussion

This study explored sexuality and sexual health in breast cancer patients and their partners, focusing on sexual satisfaction, perceived changes in satisfaction with partner sexuality before versus after diagnosis, and unmet informational needs.

Previous studies focused mainly on sexual functioning, not sexual satisfaction. A universally accepted definition or concept for measuring "sexual satisfaction" does currently not exist. Some authors assessed sexual satisfaction using a single question [39–41], whereas others utilized multi-item instruments that encompass various dimensions of sexuality [42]. In this study, both methodological approaches were applied.

A key finding concerns the discrepancy between unidimensional and multidimensional assessment of sexual satisfaction. Unidimensional, partly retrospective assessment indicated significantly lower satisfaction with partner sexuality after diagnosis compared to before. However, multidimensional item values of current sexual satisfaction were comparable to general population norms reported in literature [43]. This divergence likely reflects conceptual differences in what is being measured. While the unidimensional item captures a global evaluation of satisfaction with partner sexuality within a narrow scope, multidimensional assessment integrates broader domains, including emotional intimacy, body image, sexual functioning, and relational aspects.

Given the cross-sectional study design and the retrospective ratings, causal evidence regarding a decline in sexual satisfaction after the breast cancer diagnosis cannot be provided. Recall bias may have influenced participants’ retrospective perceptions. Nonetheless, the findings indicate that participants perceived a subjectively meaningful decline in satisfaction with partner sexuality over the course of diagnosis and treatment.

The observation that sexual satisfaction, when measured multidimensionally, remained comparable to population norms reported in literature despite the known physical and psychological burdens of breast cancer may indicate adaptive processes of redefining sexuality following illness. This interpretation is supported by findings of *Gilbert, Usher and Pelz* as well as *Loaring *et al*.* who proposed a “renegotiation” of sexuality and intimacy in the context of cancer [28, 44]. Relational intimacy has been identified as a critical dimension of sexual adjustment in couples facing cancer. The “Intimacy Process Model” developed my *Manne and Badr* proposes that emotional closeness in a relationship is an important determination of patient and partner psychological adaptation in the context of cancer [45]. In line with this framework, sexual difficulties may coexist with stable or even enhanced emotional closeness when couples are able to communicate openly and respond sensitively to each other’s needs. The present findings are compatible with this model: deficits in specific sexual domains may be offset by gains in emotional intimacy or other relational aspects. Thus, preserved sexual satisfaction does not necessarily indicate absence of distress but may represent a renegotiation of the couple’s sexuality as it was known prior to the illness.

However, this interpretation is based on comparisons with normative data reported in the literature. The findings should thus be interpreted with caution, as statistical interference would have required a matched comparison group. The results may serve as a basis for future controlled investigations.

The moderate-to-strong positive correlation between patients’ and partners’ sexual satisfaction indicates a dyadic interdependence. This finding can be situated within the “Systemic-Transactional Model of Dyadic Coping” (Bodenmann) [46], which conceptualizes stress and coping as dyadic processes involving stress communication, partner responsiveness, and joint coping. According to this model, (illness-related) stressors such as sexual changes do not remain confined to the individual but permeate the relationship system, affecting both partners’ well-being. This dyadic perspective is further supported by large-scale research using dyadic analyses. In a multinational study of over 1000 midlife couples, *Fisher *et al*.* showed that partner characteristics significantly predict sexual satisfaction and relationship happiness beyond individual factors, underscoring that sexual satisfaction is shaped by mutual relational processes rather than individual functioning alone [47].

Importantly, 75% of participants reported not having received information about sexuality in the context of breast cancer, and 64% expressed a desire for more information on this topic. This underlines a persistent unmet need for information on sexual health in breast cancer care, consistent with previous research [34]. Several concrete recommendations emerge for clinical practice. First, evidence-based, accessible educational materials – available both digitally and in print – should be developed and offered to patients and their partners at multiple points across the cancer trajectory. Such resources could help patients and partners address sexual concerns according to their individual needs. Second, clinicians should proactively, sensitively, and routinely integrate discussions of sexuality in breast cancer care. Specialized sexual-medicine workshops and training programs could support healthcare professionals in feeling confident and competent when discussing sexuality after breast cancer. Third, given the demonstrated interdependence of partners’ sexual satisfaction, couple-focused approaches may be particularly beneficial. Involving partners in counseling sessions may facilitate shared coping strategies and help reduce communication barriers within couples. Fourth, in collaboration with qualified (interdisciplinary) specialists—such as breast cancer nurses, certified sex therapists, sexual health educators, psycho-oncologists, physiotherapist—clinics could establish dedicated sexual health consultation services to provide structured support.

In conclusion, this study highlights the importance of addressing the perspectives of both patients and their partners. Partners and relationship dynamics should be considered in breast cancer care, especially in relation to sexuality and sexual health.

### Strengths and limitations

Our study offers several strengths. It applied a multimodal approach to sexuality and sexual health, addressing physiological, psychosocial, and emotional aspects based on WHO definitions. The use of validated instruments enhances reliability and validity. Our study assessed sexuality and sexual health in breast cancer patients and their “healthy” partners using elements of the EORTC QLQ-SH22. The questionnaire used in our study is applicable to both heterosexual and same-sex couples. Although ultimately no same-sex couple was represented in the study sample, the use of inclusive tools sets an important precedent for future research. Our study sample is relatively homogeneous in terms of cancer type, stage, and timing (five years post-primary treatment), enhancing internal validity.

Limitations include the low participation rate of 9% to the study invitation resulting in a small and potentially non-representative sample. Several factors likely contributed to that, including undeliverable addresses, the requirement for good German-language skills, the study inclusion criteria (especially the need for a romantic relationship in which both partners agree to participate) and the sensitivity of the topic. It is possible that some of the patients invited to the study, or their partners, were already deceased. A potential selection bias needs to be addressed. It is possible that couples with particularly open communication about sexuality may have been more likely to participate in the study. Previous studies have shown that breast cancer patients and their partners perceived open communication as crucial for their relationship and sexual health [29]. Accordingly, it may be assumed that couples in the present study, due to greater openness toward sexuality and better communication, experienced fewer problems regarding partnership, sexuality, and sexual health. Conversely, the opposite may apply: *Flynn *et al*.* reported that breast cancer patients were more likely to raise sexual issues with healthcare professionals the more pronounced their sexual problems were [33]. The direction of section bias remains uncertain, but its presence is likely, limiting the generalizability of the findings. Furthermore, there is potential response bias, as self-reported data may reflect socially desirable answers, especially on sensitive topics. It remains unclear whether partners completed the survey independently, which could influence responses. The single-center design limits generalizability to other settings. While consistent with international findings indicating unmet informational needs regarding sexuality in the context of breast cancer, the sample lacks diversity in gender identity, sexual orientation, and socioeconomic status. The study sample consisted of heterosexual, cisgender individuals with a high level of education and satisfactory financial resources. The sample is unrepresentative of same-sex couples or non-binary individuals. Although the study was open to same-sex couples, only one responded– and had to be excluded due to not meeting study inclusion criteria. Sexual und gender minority populations remain underrepresented in literature and often experience distress, as well as potential stigma and discrimination [48]. For most participants, religious beliefs had little impact on sexuality or partnership; however prior research suggests a significant influence of cultural and religious factors on sexual adjustment in breast cancer patients [49]. Thus, the findings may not be generalizable to populations with different cultural or religious backgrounds. Most participants had completed family planning. Given that fertility concerns can significantly affect the quality of life and satisfaction of breast cancer patients [50], the results may not apply to couples who still have unmet desire for children. It does not represent individuals with advanced disease. The cross-sectional design prevents assessment of temporal changes in sexual satisfaction, and the absence of baseline data limits retrospective accuracy. Finally, the use of some non-validated items reduces comparability and data reliability.

## Conclusion

This study indicates that within the present selected study sample breast cancer patients and their partners perceive a decrease in satisfaction with partner sexuality over the course of breast cancer diagnosis and treatment when assessed uni-dimensionally. Causal conclusions cannot be drawn due to the cross-sectional study design. When sexual satisfaction was assessed multidimensionally, the results demonstrated minimal deviation from general population norms reported in literature. This may reflect a renegotiation of sexuality in the context of breast cancer. The observed moderate to strong positive correlation between patients’ and partners’ sexual satisfaction underscores the importance of considering couple dynamics in breast cancer care and involving partners in the process.

Most study participants reported having received insufficient information regarding potential sexual health problems, indicating a high need for information. By addressing sexuality and sexual health physicians, psycho-oncologists, breast cancer nurses, and other health care professionals may help improve the quality of life and psychosocial adjustment of breast cancer patients and their partners. Future research should prioritize prospective, randomized controlled studies to evaluate the effectiveness of structures sexual health counseling. Such studies should investigate optimal timing for intervention and include appropriate control groups. Larger and more diverse samples, including sexual and gender minority populations, are needed. Sexuality and sexual health should be examined as multidimensional concepts in accordance with WHO definitions.

## Data Availability

The data of this study are available from the first author upon reasonable request.
